# *N*-Diphenyl­phosphanyl-*N*-{[diphen­yl(2-pyridyl­imino)-λ^5^-phosphan­yl]meth­yl}pyridin-2-amine

**DOI:** 10.1107/S1600536811037007

**Published:** 2011-09-17

**Authors:** Xiao Peng, Yan Yuan, Xue Chen

**Affiliations:** a Tianjin Kilo Pharmaceutical Sci-Tech Co. Ltd, Tianjin 300193, People’s Republic of China; b Key Laboratory of Ethnic Medicine Resourse Chemistry, Yunnan University of Nationalities, Kunming Yunnan 650031, People’s Republic of China; c College of Life Science and Technology, Kunming University, Kunming Yunnan 650214, People’s Republic of China

## Abstract

In the title compound, C_35_H_30_N_4_P_2_, the diphenyl­phosphanyl and diphen­yl(2-pyridyl­imino)-λ^5^-phosphanyl groups are attached to the central methyl C atom with a P—C—N angle of 114.09 (16)°. The mol­ecules stack along the *b* axis and inter­connect through C—H(pyrid­yl)⋯N(pyrid­yl) inter­actions, forming an infinite chain structure. The parallel chains are further inter­connected *via* C—H(benzene)⋯N(amino) and C—H(benzene)⋯π inter­actions, forming a three-dimensional framework.

## Related literature

For transition metal complexes with imino­phospho­ranyl derivatives, see: Avis *et al.* (1996 ▶, 1997 ▶). For the catalytic activity of bis­(imino­phospho­ran­yl)methane and its derivatives, see: Hill & Hitchcock (2002 ▶); Ma *et al.* (2011 ▶). For the crystal structure of an analogous compound, see: Hill & Hitchcock (2002 ▶).
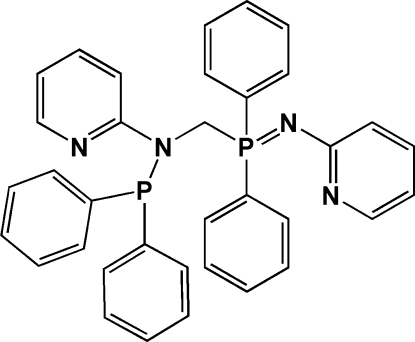


## Experimental

### 

#### Crystal data

C_35_H_30_N_4_P_2_
*M* *_r_* = 568.57
Monoclinic, 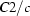

*a* = 22.505 (7) Å
*b* = 9.142 (3) Å
*c* = 29.606 (9) Å
β = 102.877 (5)°
*V* = 5938 (3) Å^3^
*Z* = 8
Mo *K*α radiation
μ = 0.18 mm^−1^
*T* = 293 K
0.40 × 0.30 × 0.20 mm

#### Data collection

Bruker APEXII CCD area-detector diffractometer
Absorption correction: multi-scan (*SADABS*; Bruker, 2007 ▶) *T*_min_ = 0.753, *T*_max_ = 1.000
16500 measured reflections
6035 independent reflections
4510 reflections with *I* > 2σ(*I*)
*R*_int_ = 0.041

#### Refinement

*R*[*F* ^2^ > 2σ(*F* ^2^)] = 0.060
*wR*(*F* ^2^) = 0.126
*S* = 1.15
6035 reflections
370 parameters
H-atom parameters constrained
Δρ_max_ = 0.39 e Å^−3^
Δρ_min_ = −0.28 e Å^−3^

### 

Data collection: *APEX2* (Bruker, 2007 ▶); cell refinement: *APEX2* and *SAINT* (Bruker, 2007 ▶); data reduction: *SAINT*; program(s) used to solve structure: *SHELXS97* (Sheldrick, 2008 ▶); program(s) used to refine structure: *SHELXL97* (Sheldrick, 2008 ▶); molecular graphics: *SHELXTL* (Sheldrick, 2008 ▶); software used to prepare material for publication: *SHELXTL* and *PLATON* (Spek, 2009 ▶).

## Supplementary Material

Crystal structure: contains datablock(s) I, global. DOI: 10.1107/S1600536811037007/ez2257sup1.cif

Structure factors: contains datablock(s) I. DOI: 10.1107/S1600536811037007/ez2257Isup2.hkl

Supplementary material file. DOI: 10.1107/S1600536811037007/ez2257Isup3.cml

Additional supplementary materials:  crystallographic information; 3D view; checkCIF report

## Figures and Tables

**Table 1:** Hydrogen-bond geometry (Å, °) *Cg* is the centroids of C7–C12 ring.

*D*—H⋯*A*	*D*—H	H⋯*A*	*D*⋯*A*	*D*—H⋯*A*
C32—H32⋯N2^i^	0.93	2.71	3.577 (3)	155
C21—H21⋯N3^ii^	0.93	2.73	3.569 (4)	150
C28—H28⋯*Cg*^iii^	0.93	2.87	3.603 (3)	136

Symmetry codes: (i) 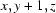; (ii) 
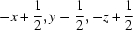
; (iii) 
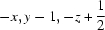
.

**Fig. 1.**
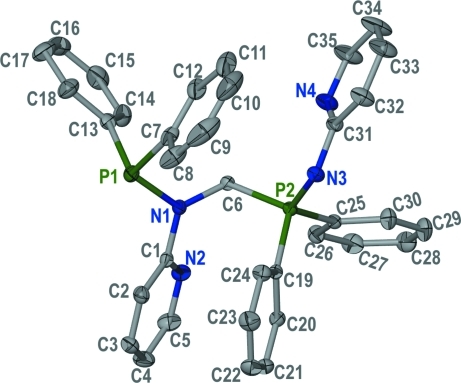
The atom-numbering scheme of the title compound. Displacement ellipsoids are drawn at the 30% probability level and H atoms are omitted for clarity.

**Fig. 2.**
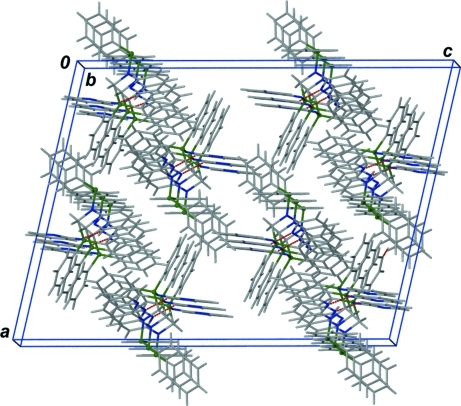
A view of the packing of the title compound. The red dashed lines represent C32—H32(pyridyl)···N2i(pyridyl) interactions that connect the molecules along the b axis (symmetry code: i: -x+1, -y, -z). The other interactions are omitted for clarity. Color codes: Green P, Blue N,Gray C.

## supplementary crystallographic information

### Comment

Bis(iminophosphorany)methane and its derivatives are attracting much attention due to their flexible coordination behavior (Avis *et al.*, 1996, 1997) and the catalytic activity of their transition metal complexes (Hill & Hitchcock, 2002; Ma *et al.*, 2011).Herein, we report the crystal structure of a mono-phosphinimine, namely ((N-2-pyridylimino)diphenylphosphoranyl)(N-2-pyridyl-N-diphenylphosphinoamino) methane.

In the crystal structure of the title compound, the (N-2-pyridylimino)diphenylphosphoranyl and the N-2-pyridyl-N-diphenylphosphinoamino groups are attached to the methyl with a P2—C6—N1 angle of 114.09 (2)°. The P2=N3 bond length of 1.593 (2) Å is comparable to that of P=N distances of 1.555 (3) and 1.573 (3) Å in bis(iminophosphorany)methane (Hill & Hitchcock, 2002). The molecules stack along the *b* axis and interconnect through C32—H32(pyridyl)···N2^i^(pyridyl) interactions (D···A 3.577 (3)Å, Table 1), forming an infinite chain. These parallel chains are further interconnected via C21—H21(benzene)··· N3^ii^(amino) and C28—H28(benzene)···Cg^iii^ interactions to form a three-dimensional framework (Cg represents the C7 to C12 benzene ring, Table 1). Symmetry codes: i: x, y+1, z; ii: x, -y-1, z-0.5; iii: -x, y-1, -z+0.5.

### Experimental

To a solution of 0.4g (0.1 mmol) N-((pyridin-2-ylamino)methyl)pyridind-2-amine in 40 ml CH_2_Cl_2_ at room temperature a solution of 0.45 g (0.2 mmol) chlorodiphenylphosphine in the presence of Et_3_N in 10 ml toluene was added dropwise, during which N_2_ gas evolved. After 2 h stirring the resultant yellow solution was evaporated, giving a white powder. The white powder was then separated and purified by column chromatography on silica gel (column of 2 cm diameter, eluent: dichloromethane/acetate = 95:5, v/v), and the title compound was obtained in 60% yield. Orange crystals of the title compound having average dimensions of 0.40 × 0.30 × 0.20 mm^3^ were obtained by slow evaporation from a solution of dichloromethane/*N,N*-dimethylformamide 1/1 (v/v).

### Refinement

The hydrogen atoms were placed in idealized positions and allowed to ride on the relevant carbon atoms, with C—H = 0.93 Å and 0.97 Å for aryl and methylene hydrogens, respectively. *U_iso_*(H) = 1.2*U_eq_*(C).

### Crystal data

**Table:**

C_35_H_30_N_4_P_2_	*F*(000) = 2384
*M**_r_* = 568.57	*D*_x_ = 1.272 Mg m^−^^3^
Monoclinic, *C*2/*c*	Mo *K*α radiation, λ = 0.71073 Å
*a* = 22.505 (7) Å	Cell parameters from 241 reflections
*b* = 9.142 (3) Å	θ = 2.1–26.3°
*c* = 29.606 (9) Å	µ = 0.18 mm^−^^1^
β = 102.877 (5)°	*T* = 293 K
*V* = 5938 (3) Å^3^	Block, orange
*Z* = 8	0.40 × 0.30 × 0.20 mm

### Data collection

**Table:**

Bruker APEXII CCD area-detector diffractometer	6035 independent reflections
Radiation source: fine-focus sealed tube	4510 reflections with *I* > 2σ(*I*)
graphite	*R*_int_ = 0.041
ω scans	θ_max_ = 26.3°, θ_min_ = 2.1°
Absorption correction: multi-scan (*SADABS*; Bruker, 2007)	*h* = −27→28
*T*_min_ = 0.753, *T*_max_ = 1.000	*k* = −11→9
16500 measured reflections	*l* = −36→33

### Refinement

**Table:**

Refinement on *F*^2^	Primary atom site location: structure-invariant direct methods
Least-squares matrix: full	Secondary atom site location: difference Fourier map
*R*[*F*^2^ > 2σ(*F*^2^)] = 0.060	Hydrogen site location: inferred from neighbouring sites
*wR*(*F*^2^) = 0.126	H-atom parameters constrained
*S* = 1.15	*w* = 1/[σ^2^(*F*_o_^2^) + (0.0394*P*)^2^ + 6.1976*P*] where *P* = (*F*_o_^2^ + 2*F*_c_^2^)/3
6035 reflections	(Δ/σ)_max_ < 0.001
370 parameters	Δρ_max_ = 0.39 e Å^−^^3^
0 restraints	Δρ_min_ = −0.28 e Å^−^^3^

### Special details

**Table:**

Geometry. All esds (except the esd in the dihedral angle between two l.s. planes) are estimated using the full covariance matrix. The cell esds are taken into account individually in the estimation of esds in distances, angles and torsion angles; correlations between esds in cell parameters are only used when they are defined by crystal symmetry. An approximate (isotropic) treatment of cell esds is used for estimating esds involving l.s. planes.
Refinement. Refinement of F^2^ against ALL reflections. The weighted R-factor wR and goodness of fit S are based on F^2^, conventional R-factors R are based on F, with F set to zero for negative F^2^. The threshold expression of F^2^ > 2sigma(F^2^) is used only for calculating R-factors(gt) etc. and is not relevant to the choice of reflections for refinement. R-factors based on F^2^ are statistically about twice as large as those based on F, and R- factors based on ALL data will be even larger.

### Fractional atomic coordinates and isotropic or equivalent isotropic displacement parameters (Å^2^)

**Table:**

	*x*	*y*	*z*	*U*_iso_*/*U*_eq_	
P1	−0.01563 (3)	0.63607 (8)	0.14930 (2)	0.03712 (18)	
P2	0.16356 (3)	0.73616 (7)	0.14753 (2)	0.02853 (16)	
N1	0.05842 (8)	0.5832 (2)	0.15430 (7)	0.0323 (5)	
N2	0.11485 (10)	0.3691 (3)	0.17411 (7)	0.0408 (5)	
N3	0.15628 (10)	0.9082 (2)	0.13974 (7)	0.0357 (5)	
N4	0.14031 (12)	0.8766 (3)	0.05916 (7)	0.0501 (6)	
C1	0.08083 (10)	0.4717 (3)	0.18710 (8)	0.0320 (6)	
C2	0.06822 (12)	0.4749 (3)	0.23102 (9)	0.0411 (6)	
H2	0.0448	0.5498	0.2393	0.049*	
C3	0.09084 (13)	0.3661 (4)	0.26186 (10)	0.0487 (7)	
H3	0.0828	0.3661	0.2914	0.058*	
C4	0.12536 (14)	0.2572 (4)	0.24880 (10)	0.0550 (8)	
H4	0.1410	0.1817	0.2690	0.066*	
C5	0.13608 (14)	0.2634 (4)	0.20499 (10)	0.0548 (8)	
H5	0.1595	0.1896	0.1961	0.066*	
C6	0.09627 (10)	0.6230 (3)	0.12172 (8)	0.0320 (6)	
H6A	0.0713	0.6761	0.0960	0.038*	
H6B	0.1101	0.5341	0.1094	0.038*	
C7	−0.01288 (12)	0.8317 (3)	0.13863 (11)	0.0451 (7)	
C8	−0.01064 (15)	0.9233 (4)	0.17666 (13)	0.0719 (11)	
H8	−0.0121	0.8834	0.2053	0.086*	
C9	−0.0062 (2)	1.0736 (5)	0.1719 (2)	0.110 (2)	
H9	−0.0043	1.1339	0.1975	0.132*	
C10	−0.00475 (19)	1.1337 (5)	0.1302 (2)	0.113 (2)	
H10	−0.0017	1.2345	0.1273	0.135*	
C11	−0.00772 (16)	1.0462 (5)	0.09285 (19)	0.0899 (14)	
H11	−0.0070	1.0877	0.0643	0.108*	
C12	−0.01182 (13)	0.8961 (4)	0.09660 (13)	0.0601 (9)	
H12	−0.0139	0.8377	0.0706	0.072*	
C13	−0.05681 (12)	0.5681 (3)	0.09276 (9)	0.0415 (6)	
C14	−0.03331 (15)	0.4781 (4)	0.06367 (11)	0.0565 (8)	
H14	0.0080	0.4557	0.0708	0.068*	
C15	−0.07070 (19)	0.4202 (4)	0.02363 (12)	0.0731 (11)	
H15	−0.0541	0.3603	0.0042	0.088*	
C16	−0.1314 (2)	0.4510 (5)	0.01285 (13)	0.0787 (12)	
H16	−0.1563	0.4120	−0.0138	0.094*	
C17	−0.15543 (17)	0.5391 (5)	0.04118 (15)	0.0806 (12)	
H17	−0.1969	0.5602	0.0338	0.097*	
C18	−0.11885 (14)	0.5978 (4)	0.08082 (12)	0.0607 (9)	
H18	−0.1360	0.6581	0.0998	0.073*	
C19	0.17852 (10)	0.7135 (3)	0.20950 (8)	0.0297 (5)	
C20	0.21447 (11)	0.6004 (3)	0.23177 (8)	0.0373 (6)	
H20	0.2324	0.5353	0.2147	0.045*	
C21	0.22379 (12)	0.5835 (4)	0.27910 (9)	0.0472 (7)	
H21	0.2477	0.5070	0.2939	0.057*	
C22	0.19752 (14)	0.6807 (4)	0.30441 (9)	0.0545 (8)	
H22	0.2040	0.6698	0.3364	0.065*	
C23	0.16185 (14)	0.7935 (4)	0.28284 (10)	0.0534 (8)	
H23	0.1442	0.8584	0.3002	0.064*	
C24	0.15210 (12)	0.8109 (3)	0.23526 (9)	0.0403 (6)	
H24	0.1280	0.8874	0.2207	0.048*	
C25	0.22768 (11)	0.6569 (3)	0.12830 (8)	0.0323 (6)	
C26	0.23091 (12)	0.5109 (3)	0.11729 (9)	0.0423 (7)	
H26	0.1993	0.4478	0.1195	0.051*	
C27	0.28149 (14)	0.4588 (4)	0.10292 (10)	0.0559 (8)	
H27	0.2831	0.3611	0.0945	0.067*	
C28	0.32934 (14)	0.5506 (4)	0.10102 (11)	0.0604 (9)	
H28	0.3637	0.5143	0.0923	0.072*	
C29	0.32629 (14)	0.6950 (5)	0.11197 (12)	0.0644 (10)	
H29	0.3586	0.7570	0.1105	0.077*	
C30	0.27536 (12)	0.7498 (4)	0.12529 (10)	0.0498 (7)	
H30	0.2732	0.8487	0.1322	0.060*	
C31	0.14809 (12)	0.9659 (3)	0.09597 (8)	0.0365 (6)	
C32	0.14870 (16)	1.1179 (3)	0.09068 (10)	0.0561 (8)	
H32	0.1532	1.1783	0.1165	0.067*	
C33	0.14268 (19)	1.1772 (4)	0.04781 (11)	0.0737 (11)	
H33	0.1427	1.2782	0.0440	0.088*	
C34	0.1366 (2)	1.0861 (4)	0.01011 (11)	0.0878 (14)	
H34	0.1333	1.1235	−0.0196	0.105*	
C35	0.1355 (2)	0.9390 (4)	0.01768 (10)	0.0770 (12)	
H35	0.1310	0.8776	−0.0079	0.092*	

### Atomic displacement parameters (Å^2^)

**Table:**

	*U*^11^	*U*^22^	*U*^33^	*U*^12^	*U*^13^	*U*^23^
P1	0.0288 (3)	0.0405 (4)	0.0426 (4)	0.0037 (3)	0.0091 (3)	0.0017 (3)
P2	0.0308 (3)	0.0285 (4)	0.0267 (3)	0.0002 (3)	0.0074 (2)	0.0001 (3)
N1	0.0263 (10)	0.0357 (13)	0.0360 (11)	0.0026 (9)	0.0094 (8)	0.0051 (9)
N2	0.0424 (12)	0.0394 (14)	0.0400 (12)	0.0099 (11)	0.0078 (10)	−0.0005 (10)
N3	0.0459 (12)	0.0303 (12)	0.0310 (11)	0.0001 (10)	0.0089 (9)	−0.0006 (9)
N4	0.0818 (18)	0.0345 (14)	0.0315 (12)	−0.0078 (13)	0.0071 (11)	−0.0018 (10)
C1	0.0263 (12)	0.0317 (14)	0.0379 (14)	−0.0039 (11)	0.0066 (10)	−0.0003 (11)
C2	0.0362 (14)	0.0461 (18)	0.0442 (15)	0.0032 (13)	0.0159 (12)	0.0044 (13)
C3	0.0500 (17)	0.056 (2)	0.0418 (16)	−0.0007 (15)	0.0136 (13)	0.0111 (14)
C4	0.0609 (19)	0.0467 (19)	0.0528 (18)	0.0083 (16)	0.0030 (15)	0.0159 (15)
C5	0.0578 (18)	0.048 (2)	0.0556 (18)	0.0198 (16)	0.0067 (14)	0.0010 (15)
C6	0.0301 (12)	0.0380 (15)	0.0281 (12)	−0.0013 (11)	0.0070 (10)	0.0008 (11)
C7	0.0306 (14)	0.0343 (16)	0.0661 (19)	0.0075 (12)	0.0013 (13)	−0.0053 (14)
C8	0.058 (2)	0.062 (2)	0.082 (2)	0.0150 (18)	−0.0128 (18)	−0.029 (2)
C9	0.079 (3)	0.064 (3)	0.156 (5)	0.021 (2)	−0.042 (3)	−0.051 (3)
C10	0.066 (3)	0.038 (2)	0.201 (6)	−0.006 (2)	−0.038 (3)	0.002 (3)
C11	0.048 (2)	0.055 (3)	0.157 (4)	0.0017 (19)	0.003 (2)	0.035 (3)
C12	0.0427 (17)	0.046 (2)	0.090 (3)	0.0077 (14)	0.0112 (16)	0.0131 (18)
C13	0.0417 (15)	0.0305 (15)	0.0494 (16)	−0.0043 (12)	0.0041 (12)	0.0082 (13)
C14	0.0552 (18)	0.050 (2)	0.063 (2)	−0.0137 (16)	0.0117 (15)	−0.0091 (16)
C15	0.099 (3)	0.059 (2)	0.060 (2)	−0.030 (2)	0.014 (2)	−0.0107 (18)
C16	0.095 (3)	0.061 (3)	0.062 (2)	−0.032 (2)	−0.020 (2)	0.013 (2)
C17	0.059 (2)	0.070 (3)	0.093 (3)	−0.007 (2)	−0.026 (2)	0.014 (2)
C18	0.0442 (17)	0.054 (2)	0.076 (2)	0.0047 (15)	−0.0049 (15)	0.0022 (17)
C19	0.0283 (12)	0.0321 (14)	0.0286 (12)	−0.0041 (10)	0.0059 (9)	−0.0005 (10)
C20	0.0334 (13)	0.0443 (17)	0.0342 (14)	0.0005 (12)	0.0073 (11)	0.0013 (12)
C21	0.0410 (15)	0.057 (2)	0.0401 (15)	−0.0001 (14)	0.0013 (12)	0.0097 (14)
C22	0.0610 (19)	0.073 (2)	0.0270 (14)	−0.0051 (18)	0.0056 (13)	0.0026 (15)
C23	0.0640 (19)	0.061 (2)	0.0374 (16)	0.0020 (17)	0.0171 (14)	−0.0099 (15)
C24	0.0469 (15)	0.0382 (16)	0.0357 (14)	0.0028 (13)	0.0088 (12)	−0.0018 (12)
C25	0.0311 (12)	0.0385 (16)	0.0284 (12)	0.0005 (11)	0.0085 (10)	0.0032 (11)
C26	0.0397 (14)	0.0426 (18)	0.0480 (16)	0.0052 (13)	0.0167 (12)	0.0013 (13)
C27	0.0609 (19)	0.053 (2)	0.0583 (19)	0.0199 (17)	0.0230 (15)	0.0017 (15)
C28	0.0475 (18)	0.085 (3)	0.0563 (19)	0.0178 (18)	0.0281 (15)	0.0078 (18)
C29	0.0432 (17)	0.084 (3)	0.073 (2)	−0.0059 (17)	0.0275 (16)	0.005 (2)
C30	0.0432 (15)	0.0495 (19)	0.0613 (19)	−0.0042 (14)	0.0211 (14)	−0.0022 (15)
C31	0.0419 (14)	0.0353 (16)	0.0309 (13)	−0.0026 (12)	0.0050 (11)	−0.0006 (11)
C32	0.097 (2)	0.0338 (17)	0.0373 (16)	−0.0079 (17)	0.0133 (16)	−0.0033 (13)
C33	0.133 (3)	0.0322 (18)	0.051 (2)	−0.006 (2)	0.010 (2)	0.0100 (15)
C34	0.167 (4)	0.055 (2)	0.0338 (18)	−0.021 (3)	0.006 (2)	0.0096 (16)
C35	0.146 (4)	0.049 (2)	0.0304 (16)	−0.021 (2)	0.0057 (18)	−0.0011 (15)

### Geometric parameters (Å, °)

**Table:**

P1—N1	1.710 (2)	C14—H14	0.9300
P1—C7	1.820 (3)	C15—C16	1.362 (6)
P1—C13	1.832 (3)	C15—H15	0.9300
P2—N3	1.593 (2)	C16—C17	1.359 (6)
P2—C19	1.802 (2)	C16—H16	0.9300
P2—C25	1.816 (2)	C17—C18	1.383 (5)
P2—C6	1.853 (2)	C17—H17	0.9300
N1—C1	1.421 (3)	C18—H18	0.9300
N1—C6	1.468 (3)	C19—C20	1.386 (3)
N2—C1	1.321 (3)	C19—C24	1.389 (3)
N2—C5	1.343 (4)	C20—C21	1.379 (4)
N3—C31	1.373 (3)	C20—H20	0.9300
N4—C35	1.336 (4)	C21—C22	1.378 (4)
N4—C31	1.342 (3)	C21—H21	0.9300
C1—C2	1.391 (3)	C22—C23	1.374 (4)
C2—C3	1.369 (4)	C22—H22	0.9300
C2—H2	0.9300	C23—C24	1.386 (4)
C3—C4	1.370 (4)	C23—H23	0.9300
C3—H3	0.9300	C24—H24	0.9300
C4—C5	1.372 (4)	C25—C26	1.379 (4)
C4—H4	0.9300	C25—C30	1.387 (4)
C5—H5	0.9300	C26—C27	1.386 (4)
C6—H6A	0.9700	C26—H26	0.9300
C6—H6B	0.9700	C27—C28	1.376 (5)
C7—C12	1.382 (4)	C27—H27	0.9300
C7—C8	1.395 (4)	C28—C29	1.365 (5)
C8—C9	1.388 (6)	C28—H28	0.9300
C8—H8	0.9300	C29—C30	1.386 (4)
C9—C10	1.359 (7)	C29—H29	0.9300
C9—H9	0.9300	C30—H30	0.9300
C10—C11	1.354 (7)	C31—C32	1.399 (4)
C10—H10	0.9300	C32—C33	1.359 (4)
C11—C12	1.382 (5)	C32—H32	0.9300
C11—H11	0.9300	C33—C34	1.375 (5)
C12—H12	0.9300	C33—H33	0.9300
C13—C14	1.379 (4)	C34—C35	1.364 (5)
C13—C18	1.389 (4)	C34—H34	0.9300
C14—C15	1.396 (4)	C35—H35	0.9300
			
N1—P1—C7	102.86 (11)	C14—C15—H15	119.9
N1—P1—C13	105.55 (12)	C17—C16—C15	119.6 (3)
C7—P1—C13	101.82 (13)	C17—C16—H16	120.2
N3—P2—C19	104.52 (11)	C15—C16—H16	120.2
N3—P2—C25	114.34 (12)	C16—C17—C18	120.7 (4)
C19—P2—C25	106.98 (11)	C16—C17—H17	119.6
N3—P2—C6	116.32 (12)	C18—C17—H17	119.6
C19—P2—C6	107.81 (11)	C17—C18—C13	121.0 (4)
C25—P2—C6	106.35 (12)	C17—C18—H18	119.5
C1—N1—C6	117.21 (19)	C13—C18—H18	119.5
C1—N1—P1	116.91 (15)	C20—C19—C24	119.5 (2)
C6—N1—P1	124.78 (16)	C20—C19—P2	121.79 (19)
C1—N2—C5	117.0 (2)	C24—C19—P2	118.66 (19)
C31—N3—P2	120.27 (18)	C21—C20—C19	120.5 (3)
C35—N4—C31	117.1 (3)	C21—C20—H20	119.8
N2—C1—C2	122.6 (2)	C19—C20—H20	119.8
N2—C1—N1	116.8 (2)	C22—C21—C20	119.6 (3)
C2—C1—N1	120.6 (2)	C22—C21—H21	120.2
C3—C2—C1	119.0 (3)	C20—C21—H21	120.2
C3—C2—H2	120.5	C23—C22—C21	120.6 (3)
C1—C2—H2	120.5	C23—C22—H22	119.7
C2—C3—C4	119.3 (3)	C21—C22—H22	119.7
C2—C3—H3	120.4	C22—C23—C24	120.2 (3)
C4—C3—H3	120.4	C22—C23—H23	119.9
C3—C4—C5	117.9 (3)	C24—C23—H23	119.9
C3—C4—H4	121.1	C23—C24—C19	119.6 (3)
C5—C4—H4	121.1	C23—C24—H24	120.2
N2—C5—C4	124.2 (3)	C19—C24—H24	120.2
N2—C5—H5	117.9	C26—C25—C30	119.7 (2)
C4—C5—H5	117.9	C26—C25—P2	123.20 (19)
N1—C6—P2	114.09 (16)	C30—C25—P2	117.1 (2)
N1—C6—H6A	108.7	C25—C26—C27	119.7 (3)
P2—C6—H6A	108.7	C25—C26—H26	120.2
N1—C6—H6B	108.7	C27—C26—H26	120.2
P2—C6—H6B	108.7	C28—C27—C26	120.5 (3)
H6A—C6—H6B	107.6	C28—C27—H27	119.8
C12—C7—C8	117.8 (3)	C26—C27—H27	119.8
C12—C7—P1	125.7 (2)	C29—C28—C27	119.9 (3)
C8—C7—P1	116.5 (3)	C29—C28—H28	120.0
C9—C8—C7	120.2 (4)	C27—C28—H28	120.0
C9—C8—H8	119.9	C28—C29—C30	120.4 (3)
C7—C8—H8	119.9	C28—C29—H29	119.8
C10—C9—C8	120.6 (5)	C30—C29—H29	119.8
C10—C9—H9	119.7	C29—C30—C25	119.8 (3)
C8—C9—H9	119.7	C29—C30—H30	120.1
C11—C10—C9	119.8 (4)	C25—C30—H30	120.1
C11—C10—H10	120.1	N4—C31—N3	119.9 (2)
C9—C10—H10	120.1	N4—C31—C32	121.1 (2)
C10—C11—C12	120.9 (5)	N3—C31—C32	119.0 (2)
C10—C11—H11	119.6	C33—C32—C31	120.0 (3)
C12—C11—H11	119.6	C33—C32—H32	120.0
C11—C12—C7	120.7 (4)	C31—C32—H32	120.0
C11—C12—H12	119.7	C32—C33—C34	119.2 (3)
C7—C12—H12	119.7	C32—C33—H33	120.4
C14—C13—C18	117.5 (3)	C34—C33—H33	120.4
C14—C13—P1	125.9 (2)	C35—C34—C33	117.8 (3)
C18—C13—P1	116.2 (2)	C35—C34—H34	121.1
C13—C14—C15	120.9 (3)	C33—C34—H34	121.1
C13—C14—H14	119.5	N4—C35—C34	124.8 (3)
C15—C14—H14	119.5	N4—C35—H35	117.6
C16—C15—C14	120.3 (4)	C34—C35—H35	117.6
C16—C15—H15	119.9		
			
C7—P1—N1—C1	143.92 (19)	C14—C15—C16—C17	0.3 (6)
C13—P1—N1—C1	−109.72 (19)	C15—C16—C17—C18	0.0 (6)
C7—P1—N1—C6	−48.4 (2)	C16—C17—C18—C13	−0.2 (6)
C13—P1—N1—C6	58.0 (2)	C14—C13—C18—C17	0.0 (5)
C19—P2—N3—C31	175.99 (19)	P1—C13—C18—C17	−173.4 (3)
C25—P2—N3—C31	59.4 (2)	N3—P2—C19—C20	−148.8 (2)
C6—P2—N3—C31	−65.3 (2)	C25—P2—C19—C20	−27.2 (2)
C5—N2—C1—C2	1.7 (4)	C6—P2—C19—C20	86.8 (2)
C5—N2—C1—N1	−179.9 (2)	N3—P2—C19—C24	32.5 (2)
C6—N1—C1—N2	−31.0 (3)	C25—P2—C19—C24	154.1 (2)
P1—N1—C1—N2	137.68 (19)	C6—P2—C19—C24	−91.9 (2)
C6—N1—C1—C2	147.4 (2)	C24—C19—C20—C21	0.4 (4)
P1—N1—C1—C2	−43.9 (3)	P2—C19—C20—C21	−178.3 (2)
N2—C1—C2—C3	−1.3 (4)	C19—C20—C21—C22	−0.5 (4)
N1—C1—C2—C3	−179.6 (2)	C20—C21—C22—C23	0.4 (5)
C1—C2—C3—C4	0.2 (4)	C21—C22—C23—C24	−0.2 (5)
C2—C3—C4—C5	0.5 (5)	C22—C23—C24—C19	0.1 (4)
C1—N2—C5—C4	−1.0 (5)	C20—C19—C24—C23	−0.2 (4)
C3—C4—C5—N2	−0.1 (5)	P2—C19—C24—C23	178.5 (2)
C1—N1—C6—P2	−75.7 (3)	N3—P2—C25—C26	−157.5 (2)
P1—N1—C6—P2	116.67 (18)	C19—P2—C25—C26	87.3 (2)
N3—P2—C6—N1	−97.21 (19)	C6—P2—C25—C26	−27.7 (2)
C19—P2—C6—N1	19.7 (2)	N3—P2—C25—C30	23.1 (2)
C25—P2—C6—N1	134.16 (18)	C19—P2—C25—C30	−92.1 (2)
N1—P1—C7—C12	79.6 (3)	C6—P2—C25—C30	152.9 (2)
C13—P1—C7—C12	−29.6 (3)	C30—C25—C26—C27	−0.6 (4)
N1—P1—C7—C8	−99.3 (2)	P2—C25—C26—C27	−179.9 (2)
C13—P1—C7—C8	151.5 (2)	C25—C26—C27—C28	2.1 (4)
C12—C7—C8—C9	−1.3 (5)	C26—C27—C28—C29	−2.0 (5)
P1—C7—C8—C9	177.7 (3)	C27—C28—C29—C30	0.4 (5)
C7—C8—C9—C10	0.8 (6)	C28—C29—C30—C25	1.2 (5)
C8—C9—C10—C11	0.1 (7)	C26—C25—C30—C29	−1.0 (4)
C9—C10—C11—C12	−0.5 (6)	P2—C25—C30—C29	178.3 (2)
C10—C11—C12—C7	−0.1 (5)	C35—N4—C31—N3	−176.9 (3)
C8—C7—C12—C11	1.0 (4)	C35—N4—C31—C32	2.4 (5)
P1—C7—C12—C11	−177.9 (2)	P2—N3—C31—N4	6.4 (3)
N1—P1—C13—C14	7.1 (3)	P2—N3—C31—C32	−172.9 (2)
C7—P1—C13—C14	114.2 (3)	N4—C31—C32—C33	−1.4 (5)
N1—P1—C13—C18	179.8 (2)	N3—C31—C32—C33	177.8 (3)
C7—P1—C13—C18	−73.1 (3)	C31—C32—C33—C34	−0.5 (6)
C18—C13—C14—C15	0.4 (5)	C32—C33—C34—C35	1.4 (7)
P1—C13—C14—C15	173.0 (2)	C31—N4—C35—C34	−1.5 (6)
C13—C14—C15—C16	−0.5 (5)	C33—C34—C35—N4	−0.4 (7)

### Hydrogen-bond geometry (Å, °)

**Table:**

Cg is the centroids of C7–C12 ring.

**Table:**

*D*—H···*A*	*D*—H	H···*A*	*D*···*A*	*D*—H···*A*
C32—H32···N2^i^	0.93	2.71	3.577 (3)	155.
C21—H21···N3^ii^	0.93	2.73	3.569 (4)	150.
C28—H28···Cg^iii^	0.93	2.87	3.603 (3)	136.

Symmetry codes: (i) *x*, *y*+1, *z*; (ii) −*x*+1/2, *y*−1/2, −*z*+1/2; (iii) −*x*, *y*−1, −*z*+1/2.
